# Death from Hæmorrhage Following the Extraction of a Tooth

**Published:** 1869-03

**Authors:** O. Salomon


### ARTICLE IV.

### *Death from Hæmorrhage Following the Extraction of a Tooth.*

Translated from the German of Dr. Neimeyer, by Mr. O. Salomon,

Although some instances of death from hæmorrhage, caused by the extraction of teeth have been published, this statement will perhaps not be uninteresting, since among 9442 teeth which were extracted in the Hospital of the Grand Duchy of Brawnschweig during the period embraced from 1859 to 1866, this is the only instance in which death ensued from hæmorrhage.

Although in the last few years dissections are prosecut. ed with greater particularity and care, and Pathological Anatomy, Microscopy and Physiology have made decided advancement, it has not yet been clearly demonstrated whether the hæmorrhagic diathesis is owing to the condition of the blood itself or the vein and artery coats.

Some close observers believe and advance these last opinions, as in many dissections the coats of the veins and arteries have been found very thin and loose ; whether this is the only course must yet be discovered as there are others again who entertain a contrary opinion.

Even obscure as the cause of the Hæmophilie are, we are equally ignorant, in many cases with regard to the therapeutics, as the following case demonstrates, in which, though receiving the benefit of the most skillful attention, death ensued.

On the 8th of May, 1864 at 1 15 P. M. a Mr. L. K. from L. aged 21 years and a tailor by trade, arrived at the Ambulatorische Clinic "of Mr." Medicinalrath "Uhde" for the purpose of having a back tooth on the left side of the inferior maxillary extracted which was decayed, and had given him severe pain for several days and nights. The operation was very easy and the patient immediately relieved from pain.

The bleeding was slight though continuing somewhat longer than is usual in cases of teeth extraction, and the patient had to take cold water with alumen crudum in his mouth after which the bleeding in a measure ceased. Patient stated that previous to this he had quite severe hæmorrhages caused by very trifling incisions. His father and brother are also reported having suffered severe hæmorrhages from very slight scratches.

Mr. K. was well developed for his age, had a pale complexion, soft skin, blonde hair, blue eyes and colorless or pale mucous membrane; and with the exception of the aforementioned hæmorrhages, always had good health.

He left the Hospital at 5 P. M. at which time the bleeding had ceased to such an extent that it only discolored the saliva slightly.

On the following morning at 6 o'clock the patient was brought in a carriage to the Surgical Clinic. His companions stated that there was no bleeding on the previous evening from 8 to 10 o'clock; from this hour however until his arrival in the hospital the patient lost a great quantity of blood. He was wholly anæmic the face was pale, extremities cold, pulse barely perceptible, often recurring of vomiting, hiccup, eyes fixed, he could neither walk nor stand, and could with the greatest difficulty articulate a few words the bleeding from the alveolar cavity being so profuse.

Patient was immediately placed in bed, the coagulated blood removed from the mouth, and the alveolar cavity filled with a small quantity of cotton prepared with acidum tannicum and alumen crudum. This however did not have the desired effect until a conical shaped cork had been pressed firmly on the top of the cotton.

The extracted tooth could not in this case be used as a tampon, as the patient had lost it. As diet, the patient received beef tea, coffee and wine; his recovery was quite rapid and after four days, on the 11th of May, there having been no recurrence of the hæmorrhage he left the hospital.

On the following evening at 10 o'clock the patient came again to the hospital having suffered severe hæmorrhages since the morning, which commenced immediately after removing the tampon, on account of its being uncomfortable, which was done.

A new and similar shaped tampon was immediately made and applied, but without success; even Liq. ferri sesqui chlorati with lint did not stop it, and therefore a small piece of nitrate of silver was placed in the alveolar cavity and the cork placed on it. Inwardly, acid Halleri and abstinence from all warm drinks and food.

The bleeding did not cease after this treatment, and as a last resource the actual cautery had to be applied which, after several applications followed by the tampon, had the desired effect.

The patient became through this loss of blood again wholly anæmic. The pulse barely perceptible, 132 strokes per minute, extremities cold, temperature in the arm pits 32. 7° C., respiration 36.

Inwardly—R. Tinct. ferri acet. ʒ ij.

Aq. dest. ℥ ij.

S.—1 table spoonful every hour.

On the 15th of May gaining strength, pulse 120, temperature 34°, respiration 24. On the 19th of May the patient against all remonstrances removed the tampon, whereupon the bleeding immediately commenced with all its former violence, and was only stopped after six hours application of tampon and hot irons.

All the strength which had been gained was again lost by this hæmorrhage.

Patient revived again, pulse almost imperceptible, temperature 33° C.; extremities cold and swollen, face pale, eyes fixed; respiration became slower, throat rattle set in and patient died anæmic on the 24th at 6 o'clock A. M.

Dissection was made 18 hours after death had ensued. The body had lost very little flesh, œdematous, swollen; the stiffness was not remarkable, and hardly any spots could be found on the back. The brain appeared very white and the gray substance could hardly be observed; all the sinuses, as also, the plexus choroidei contained barely a trace of blood, and in the ventricles could only be found a small quantity of cerebro-spinal liquid. At the place where the tooth had been extracted nothing abnormal could be seen on dissection.

The lungs adhered at seven places with the pleura costalis the pericardium and diaphagm through slight adhesion overgrown; out of an incision made in the lungs a bloody watery liquid commenced to flow. On the left branch of the trachea a bronchial gland of the size of a filbert was found covered with lime. The pericardium with the front side of the head was combined in a jelly-like mass and contained only a small quantity of Liq. pericardii. The heart was somewhat enlarged. The muscles appeared very pale, and under the microscope appeared a fatty degeneration of the muscular tissues. In the ventricles only a very small quantity of coagulated blood was found. In the auricles and ventricles nothing abnormal. The vein and artery coats appeared in some places unusually thin, transparent and weak. The blood was rather watery, contained only a small quantity of red blood corpuscles. In the abdomen only slight exudation. The liver was considerably enlarged, had a whitish-yellow appearance, and under the microscope free or loose particles of fat could be seen in the liver cells as well as in their vicinity. The gall bladder contained only a small quantity of yellowish green gall. The spleen had a grayish red color, was very soft and quite large. On the kidneys nothing noteworthy was to be found except that the right was somewhat darker than the left. The mucous membrane of the whole of the intestines, as also of the bladder, appeared very pale.

We are forced to accept the lesson contained herein, for we as dentists are always liable to these accidents, there being nothing to indicate or discover the defect, except the statements from the patient which are not always reliable. Therefore, nothing remains for us to do in the premises except to observe carefully the extent of the bleeding after extraction, and to warn the patient in case of renewed bleeding to return at once.

In the foregoing statement it is not mentioned which tooth was extracted, the name (the back tooth) could be easily construed as indicating the 1st molar, the physicians in general not regarding it as important.

The name "Wisdom Tooth" is too familiarly known to be designated otherwise if it was the 3d molar that was here meant. Without seeking to differ as to the correctness of the treatment pursued in this case, considering the persistency of the hæmorrhage and the inconvenience of the patient, it might have been advisable to have a dentist take the impression of the adjoining teeth and insert an appliance which would have held the tampon in its place, and at the same time allowed the patient free use of the tongue and mouth.

---

